# Young adult and middle age mortality in Butajira demographic surveillance site, Ethiopia: lifestyle, gender and household economy

**DOI:** 10.1186/1471-2458-8-268

**Published:** 2008-07-31

**Authors:** Mesganaw Fantahun, Yemane Berhane, Ulf Högberg, Stig Wall, Peter Byass

**Affiliations:** 1 Department of Community Health, Addis Ababa University, PO Box 24762, Code 1000, Addis Ababa, Ethiopia; 2 Umeå International School of Public Health, Epidemiology and Public Health Sciences, Umeå University, Umeå, Sweden; 3 Department of Obstetrics and Gynaecology, Umeå University, Umeå, Sweden

## Abstract

**Background:**

Public health research characterising the course of life through the middle age in developing societies is scarce. The aim of this study is to explore patterns of adult (15–64 years) mortality in an Ethiopian population over time, by gender, urban or rural lifestyle, causes of death and in relation to household economic status and decision-making.

**Methods:**

The study was conducted in Butajira Demographic Surveillance Site (DSS) in south-central Ethiopia among adults 15–64 years old. Cohort analysis of surveillance data was conducted for the years 1987–2004 complemented by a prospective case-referent (case control) study over two years.

Rate ratios were computed to assess the relationships between mortality and background variables using a Poisson regression model. In the case-referent component, odds ratios (95% confidence intervals) were used to assess the effect of certain risk factors that were not included in the surveillance system.

**Results:**

A total of 367 940 person years were observed in a period of 18 years, in which 2 860 deaths occurred. One hundred sixty two cases and 486 matched for age, sex and place of residence controls were included in the case referent (case control) study. Only a modest downward trend in adult mortality was seen over the 18 year period. Rural lifestyle carried a significant survival disadvantage [mortality rate ratio 1.62 (95% CI 1.44 to 1.82), adjusted for gender, period and age group], while the overall effects of gender were negligible. Communicable disease mortality was appreciably higher in rural areas [rate ratio 2.05 (95% CI 1.73 to 2.44), adjusted for gender, age group and period]. Higher mortality was associated with a lack of literacy in a household, poor economic status and lack of women's decision making.

**Conclusion:**

A complex pattern of adult mortality prevails, still influenced by war, famine and communicable diseases. Individual factors such as a lack of education, low economic status and social disadvantage all contribute to increased risks of mortality.

## Background

The health problems of adults have been neglected in many developing countries and a relatively small proportion of public health research in developing societies has been devoted to characterising the normal course of life through middle age. This is related to the fact that childhood mortality is high and the common assumption that once the period of childhood is survived, the survival disadvantage is small [1]. However, studies in several developing countries have shown high rates of premature mortality in adults. The risk of a 15-year-old person dying before reaching 60 years of age is 25% for men and 22% for women in developing countries, more than double than that in the developed world, where the respective figures are 12% and 5 % [1].

On the other hand, developing countries are a very heterogeneous group in terms of mortality. A contrast between low-mortality developing countries such as China (with more than one-sixth of the world's population) and high-mortality countries in Africa (with one-tenth of the global population) illustrates the extreme diversity in health conditions among developing countries. Less than 10% of deaths in China occur below 5 years of age compared with 40% in Africa. Conversely, 48% of deaths in China occur beyond age 70, compared with only 10% in Africa [2]. In addition to the high and neglected problem of adult death which needs attention on its own, the health of adults is essential for the wellbeing of the young and the elderly.

Thus the need to understand the trend and causes of adult mortality to develop national and international policies can not be undermined [3].

Lifestyle differentials in sub-Saharan Africa largely revolve around the distinction between village life, in which households exist primarily in agricultural subsistence economies, and more urbanised lifestyles in small market towns and particular sectors of larger cities, where households do not have appreciable land and people generally live in extremely poor cash-based economies. Although this is frequently characterised as a rural-urban divide, this can be misleading in global terms and needs to be understood in its own context.

In high and middle income countries, mortality among adult females is generally lower than for their male counterparts. However, in low income countries gender differences in mortality tend to be smaller and national averages may hide important variations. Stagnating or increasing adult mortality has recently been reported from sub-Saharan Africa [4], although this may be largely due to the HIV epidemic in the region. The recently introduced Gender Gap Index, measuring economic participation, educational attainment, health and survival and political empowerment shows very low scores, consistent with female disadvantage, in many developing countries [5]. This may be associated with higher female mortality in such settings. Ethiopia ranks 100 out of 115 countries in the Gender Gap Index [5]. Heavy workload, lack of access to health services, poverty, poor social status and decision making power are among highly prevalent factors that may be detrimental to the health of women in south central Ethiopia [6].

Poverty is extremely difficult to measure and understand in societies where asset ownership is often minimal, and confounded by differences in poor rural and poor urban lifestyles, such as differential access to health facilities. Social capital, in addition to material wealth, is increasingly being recognised as significant dimension to well-being among the poor [7]

A complex epidemiological transition has been characterised in the Butajira Rural Health Program (BRHP) for the period 1987–2004, showing an overall mortality decline, but least so for some adult age groups [8]. Illiteracy has previously been shown as a mortality risk factor [9] closely linked to poor socio-economic conditions [10].

Our objective here is to describe and assess adult mortality differentials in the Butajira area, exploring differences and trends associated with rural and urban lifestyles, among men and women, and according to material and social poverty.

## Methods

The study was conducted in the Butajira Rural Health Programme (BRHP) Demographic Surveillance Site (DSS). This site is located in the Butajira District, some 130 km to the south of Addis Ababa. Ethiopia is divided administratively into small administrative units called *kebeles*, each containing a few thousand people. The DSS consists of nine rural and one urban *kebeles *sampled in 1986 using a probability proportionate to size technique. Census done in 1986 was followed by first, monthly then (since 1999) quarterly surveillance of vital events (births, deaths, marriage and migration) and periodic census for quality control and updates of socio-demographic characteristics of the study population [11].

All deaths have since been recorded in household visits. Cause of death as reported by a close relative or other household informant was recorded throughout. Since mid-2003, more rigorous verbal autopsy interviews have been applied using a questionnaire based on a WHO-INDEPTH instrument [12,13].

This study is based on the analysis of two datasets. Firstly, the longitudinal surveillance of the open population cohort during the period 1987–2004 allowed analyses of mortality patterns and trends among adults aged 15–64 years by urban or rural residence, gender and background factors (presence of at least one literate person in the household and house ownership). A total of 367 940 person years were observed in a period of 18 years, in which 2 860 deaths occurred, amounting to a crude mortality rate of 7.8 per 1000 person years. Secondly, a prospective case-referent (case control) study on deaths during a two-year period allowed analyses on determinants of mortality, focusing particularly on issues related to women's status (household decision making, social capital and economic status). Items for inclusion into these measures were identified through a consensus process reached with community representatives based on instruments used in the Ethiopian Demographic and Health Survey [14] and a World Bank report [15].

Household decision making was assessed by using four questions about who makes decision among household members including big decisions (decisions to change place of residence, buy, sell or reconstruct a house, rent land, etc.); routine household decisions that include decisions on buying and selling food items and day-to-day activities in the household; decisions to visit family and friends; and decisions to take a sick family member to a health institution. Consensus items for social capital in the area included the ability to borrow money in case of need, membership of the *Kebele *(smallest administrative unit) leadership, and membership of community organizations, trusting people, and thinking that people can hurt. Similarly, community discussants reached consensus about the scoring and relevance to the area items among those stipulated by the Ethiopian demographic and health survey. The details are presented elsewhere [16].

One hundred sixty two of the recorded 169 deaths that occurred in the period August 2003 to July 2005 inclusive were prospectively (hence, the name prospective case referent) included in the study. For every case, three referents were randomly selected matched for age, sex and place of residence.

Data were collected by trained interviewers who had completed high school and had previous experience in community-based data collection. For the case referent component, interviews took place from 45 to 60 days after death in most cases. Forty-five days is regarded as the usual mourning period in the study area. The 60-day maximum was applied to minimize recall bias concerning details of symptoms and circumstances of death.

The reported causes of death were grouped as communicable (including maternal) (e.g. diarrhoea, malaria, TB) and non-communicable (e.g. heart disease, injury, sudden death). The unknown and "other" categories were included as non-communicable causes on the basis that people in these communities are more familiar with, and find it easier to recognise, classic communicable causes of death.

Age groups for analysis were taken as 15 to 44 years (younger adulthood, including the reproductive period for women) and 45 to 64 years (older adulthood).

Surveillance data were analysed using the Cohort software (Umeå University) and STATA 9. Crude and adjusted rate ratios and their 95% confidence intervals were computed to assess the relationships between mortality and background variables using a Poisson regression model, in which person-time under surveillance was the rate multiplier and standard errors were adjusted for clustering at the *kebele *level.

Case-referent data were entered using SPSS Version XI and matched case-referent analyses were conducted. Odds ratios and their 95% confidence intervals were used to assess effects of risk factors on mortality.

This study was ethically approved by the Ethiopian National Ethical Clearance Committee as part of the BRHP research activities and individual informed consent was obtained from each participant.

## Results

**Figure 1:**
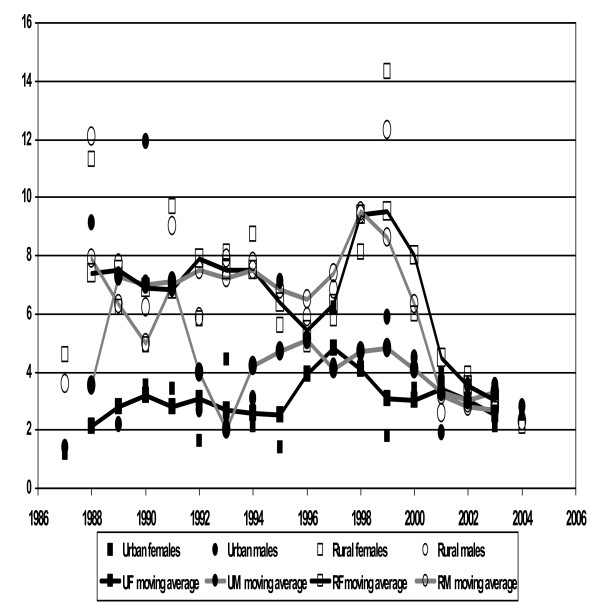
**Three year moving averages of mortality rates (per 1000 person-years) among adults 15–44**. Mortality rate per 1000 years and superimposed three years moving average mortality rate graph among adults 15–44 yrs Butajira, Ethiopia 1987–2004.

**Figure 2:**
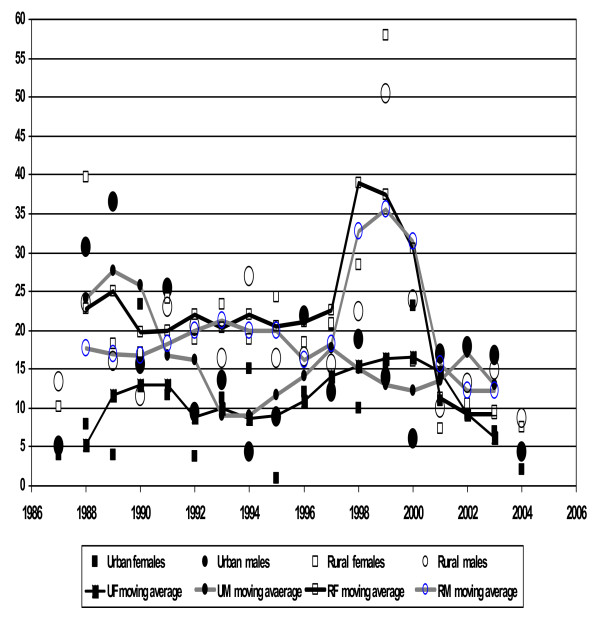
**Three year moving averages of mortality rates (per 1000 person-years) among adults 45 – 64**. Mortality rates per 1000 person years and superimposed three year moving averages graph among adults 45–64 years, Butajira, Ethiopia 1987–2004.

**Figure 3:**
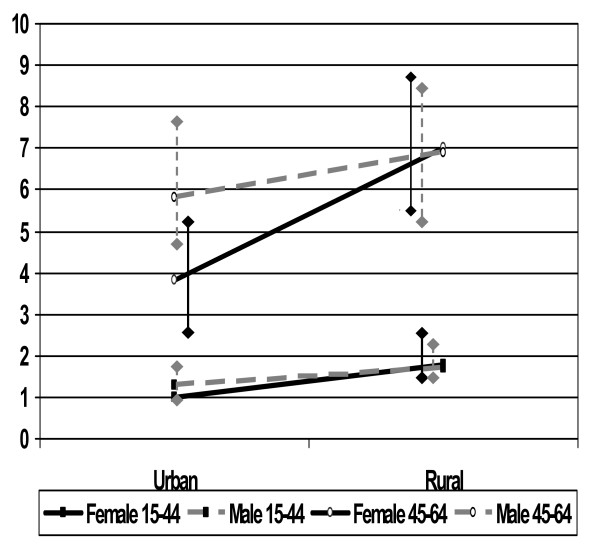
**Adjusted mortality rate ratios (reference group urban females 15–44 yr), Butajira**. Adjusted for time period mortality rate ratios (reference group urban females 15–44 yr), Butajira, Ethiopia (vertical bars represent 95% confidence intervals) by sex, age group and place of residence.

The complex patterns of mortality during the period 1987–2004 are shown as mortality rates per 1000 person years and 3-year moving averages in Figures 1 and 2, for the 15–44 and 45–64 year age groups respectively. Table 1 shows mortality rates by age group, gender, period and area. There was a modest downward trend in adjusted mortality over the 18-year period, with 1993–98 at 93% of the initial mortality (95% CI 84% to 102%), and 1999–2004 at 73% (95% CI 67% to 81%) (adjusted for age group, area and gender), but behind these trends substantial epidemic peaks were observed. Considerable excess male mortality was evident in the urban area up to 1991, and the rural area experienced a substantial peak in mortality from 1998 to 2000. These phenomena contributed substantially to the overall higher male mortality rate in the urban area [rate ratio 1.42 (95% CI 1.15 to 1.77), adjusted for age group and period] and the overall higher mortality in the rural area [rate ratio 1.62 (95% CI 1.44 to 1.82), adjusted for gender, age group and period]. There was no significant overall association between gender and mortality. Apart from the effect of age, the most evident influence on mortality was the divide between urban and rural lifestyles, with consistently higher mortality for rural dwellers [rate ratio 1.62 (95% CI 1.44 to 1.82), after adjusting for gender, period and age group], as shown further in Figure 3.

**Table 1:** Mortality rate ratios (95% confidence intervals) in relation to age group, residence, time and gender; Butajira 1987–2004

parameter	Level	Unadjusted rate ratio (95% CI)	adjusted rate ratio (95% CI)
age group	15–44 years	1.00	1.00
	45–64 years	3.38 (3.14 to 3.64)	3.30 (3.06 to 3.55)
gender	Female	1.00	1.00
	Male	1.05 (0.98 to 1.13)	1.03 (0.95 to 1.10)
period	1987–1992	1.00	1.00
	1993–1998	0.95 (0.86 to 1.04)	0.92 (0.84 to 1.02)
	1998–2004	0.71 (0.64 to 0.78)	0.73 (0.67 to 0.81)
area	Urban	1.00	1.00
	Rural	1.79 (1.60 to 2.02)	1.62 (1.44 to 1.82)

The overall effect of no literate person in a household carried a mortality rate ratio of 2.38 (95% CI 2.19 to 2.59, adjusted for area, gender, age group and period), and was particularly seen in rural areas [rate ratio 3.16 (95% CI 2.92 to 3.41)] and among men [rate ratio 3.19 (2.88 to 3.54)]. Non-ownership of the house where people lived was strongly associated with mortality [rate ratio 1.77 (95% CI 1.54 to 2.04), adjusted for area, gender, age group and period] and was seen particularly in rural areas [rate ratio 3.23 (95% CI 2.99 to 3.49)].

Of the 2,855 deaths, 1,513 (53%) were attributed to communicable diseases and the remaining 1,342 (47%) to non communicable diseases (NCD). Overall, males had slightly higher rates of NCD mortality than females [rate ratio 1.14 (95% CI 1.03, 1.27), adjusted for age group, period and area] and this effect was stronger in the urban area [rate ratio 1.65 (95% CI 1.24 to 2.21)]. Communicable disease mortality did not differ significantly by gender, but was substantially higher in the rural areas [rate ratio 2.05 (95% CI 1.73 to 2.44), adjusted for gender, age group and period].

Analyses of the case-referent data for the years 2003–2005 showed that marital status, lower household economic status and weaker joint household decision making were significantly associated with adult mortality. Marital status was strongly associated with male mortality in the age group 15–44. Unmarried men (single, divorced and widowed) were 3 times more likely to die [odds ratio 3.56 (95% CI 1.86, 6.83)] compared to the married. The differences were not statistically significant among females, nor in the age groups 45–64. Lower household economic status was strongly associated with mortality in both males and females in rural areas. In the urban area, economic status had a greater effect on female mortality (Table 2). Lack of joint household decision making was strongly associated with female mortality in rural areas [OR 1.93(95% CI 1.11, 3.36)], but not in urban areas.

**Table 2:** Adjusted odds ratios (95% confidence intervals) for mortality in relation to literacy, economic status and household decision making, Butajira, 2003 – 2005

Variables			**Males**			**females**		
			
			**cases ** n (%)	**referents ** n (%)	**adjusted OR ** (95%CI)	**cases ** n (%)	**referents ** n (%)	**adjusted OR ** (95%CI)
Literacy	urban	able to read and write	8 (38)	39 (60)	1.00	7 (39)	21 (38)	1.00
		unable to read and write	13 (62)	26 (40)	1.86 (0.60,5.87)	11 (61)	34 (62)	0.75 (0.27,2.09)
	rural	able to read and write	17 (27)	58 (31)	1.00	5 (9)	26 (15)	1.00
		unable to read and write	46 (73)	129 (69)	0.97 (0.53,1.75)	54 (91)	150 (85)	1.20 (0.43,1.35)
Household economic status	urban	higher	9 (43)	37 (57)	1.00	6 (33)	40 (74)	1.00
		Lower	12 (57)	28 (43)	1.50 (0.50, 4.49)	12 (67)	14 (26)	3.40 (1.24,9.32)*
	rural	higher	32 (51)	137 (73)	1.00	35 (59)	144 (82)	1.00
		Lower	31 (49)	50 (27)	1.99 (1.18,3.36)*	24 (41)	32 (18)	1.90 (1.08,3.34)*
Joint household decision making	urban	stronger	16 (76)	53 (81)	1.00	10 (56)	39 (71)	1.00
		Weaker	5 (24)	12 (19)	1.14 (0.26,5.14)	8 (44)	**16 (29)**	1.64 (0.60,4.44)
	rural	stronger	40 (64)	141 (75)	1.00	31 (53)	135 (76)	1.00
		weaker	23 (36)	46 (25)	1.46 (0.84,2.54)	28 (47)	41 (24)	1.93 (1.11,3.36)*

For women, participation in routine household decision making and decisions to visit relatives and health facilities was strongly associated with mortality (Table 3), while factors such as ability to borrow money in case of need, membership of the *kebele *leadership, membership of community organizations, trusting people, thinking that people can hurt were not significantly associated with mortality.

**Table 3:** Odds ratios (95% confidence intervals) for mortality in relation to decision making, with adjustment for economic status, among adult women in rural communities, Butajira, 2003–2005

**Type of household decision making**		**cases n (%)**	**referents n (%)**	**adjusted OR (95%CI)**
woman makes "big" decisions	yes	31 (53)	120 (68)	1.00
	no	28 (47)	56 (32)	1.65 (0.96,2.85)
woman makes routine decisions	yes	36 (61)	153 (87)	1.00
	no	23 (39)	23 (13)	2.00 (1.12, 3.56)
woman makes decisions to visit relatives and friends	yes	30 (51)	138 (78)	1.00
	no	29 (49)	38 (22)	1.97 (1.13,3.40)
woman makes decisions to visit health facility	yes	29 (49)	133 (76)	1.00
	no	30 (51)	43 (24)	1.83 (1.05,3.17)

## Discussion

The overall picture of adult mortality in this area has to be contextualised in terms of local circumstances and effects. There was no very clear trend in mortality over the entire 18-year period, but very large and specific effects were noted during the course of the study. In the first few years, the very considerable excess male mortality in the urban area was likely associated with the final years of the Ethiopian civil war. Although the Butajira area was not heavily militarised, there were high rates of somewhat random conscription of adult males to conflict areas, which was easier to implement from higher-density urban areas. There were 50% more women than men in the 25–34 year age group resident in Butajira at that time, which gives some indication of the extent of the conscription effect and makes it plausible that the observed excess in male mortality at that time could be largely due to the war. This in turn makes it difficult to interpret longer term gender effects on mortality.

The second major effect on mortality, this time affecting primarily rural areas, was a period of unusual rainfall in 1998–2000 which led to widespread food shortages and consequent epidemics. This has been described in detail elsewhere [17]. This also complicates the overall interpretation of mortality rates, particularly in respect of the figures at the end of the period. Subsequent fall in rates may partly be due to a shadow effect of premature mortality during the epidemic period, although this was at the same time as a new hospital coming on-stream in Butajira.

Notwithstanding these important specific mortality phenomena, the effects of urban and rural lifestyles, gender and relative poverty can be considered in relation to mortality. As has consistently been the case in previous analyses from Butajira [6,9] there is a mortality disadvantage associated with rural living which overrides age and gender effects. The precise determinants of this effect remain to some extent unclear, since distance to the town, access to health facilities, lack of a cash economy and differential availability of food are all highly confounded within the urban-rural paradigm. The fact that the 1998–2000 rainfall and food supply phenomena discriminated against those having a rural lifestyle, even in the case of villages situated very close to the town, emphasises the urban-rural divide.

In terms of gender, adult mortality differences were minimal, particularly if the apparent war-time effects on male mortality are discounted. Butajira district still experiences relatively low levels of HIV prevalence compared with other locations in sub-Saharan Africa, and so HIV-related mortality is probably still too small as a proportion of total mortality to have any appreciable overall effect. The relatively higher rural female mortality where there was no joint household decision making pinpoints the effect of lower status for rural women [6]. Decision making is a key indicator of women's status and interventions to empower women have shown great impact on women's quality of life, autonomy and authority, and improved infant and maternal survival [7,18].

Poverty – whether in terms of assets, education or social capital – seems to be an important determinant of mortality. Education has been previously associated with improved survival in the area [9,10]. Absence of a literate person in a household appears to particularly affect mortality among males and in rural areas. It is possible that presence of a literate person is more advantageous to men than to women in terms of improving awareness from which men, who are generally the decision makers, may be benefiting to a greater extent. This issue needs to be explored further.

Adjusted for other factors, house ownership affected rural residents rather than urban residents, although the proportion of residents in rural areas who do not own the house they live in is much smaller (about 5%). Rural houses in Butajira are generally small huts [11]. Thus residents in rural areas who do not own houses could be a particularly disadvantaged group, socially and economically. By contrast, the urban lifestyle in Butajira town for many people amounts to renting a basic room or part of a house, implying that urban non-owners are part of a cash economy and may be very different from rural non-owners.

A limitation of the surveillance data is that not all important predictors of mortality were included in regular data collection and some parameters, such as cause of death, were imprecise. Thus classification between communicable and non-communicable diseases was based on the responses to simplified questions and probably introduced some misclassification bias. In addition, some missing data on literacy and marital status may have also introduced some bias.

A limitation of the case referent component is the potential for recall bias and possible gaps in information on the deceased by respondents, although the study was conducted within a reasonable period of time [19]. Factors that were used to assess social capital, for example, did not show differences between the cases and referents and this may be related to respondents' lack of accurate information about the deceased concerning these issues. In addition, the sample size did not allow comparisons of mortality effects in sub-groups.

By comparison with a previous study [20], there appears a higher occurrence of non communicable disease, indicating the increasing double burden of communicable and non communicable diseases. Urban-rural differences in non-communicable and communicable diseases may have been obscured by opposing influences of increases in non-communicable diseases and HIV/AIDS [21]. Thus, while deaths due to communicable diseases remain high, indicating a relatively early stage of epidemiological transition, increases in NCDs and HIV/AIDS would indicate a need for preparedness to deal with this triple burden.

Being unmarried was strongly associated with mortality among urban males. This was not true for other females and males in rural areas. That mortality rates are lower for married individuals has long been documented [22], which could be due to selection or protection. Excess mortality seen among the unmarried in rural Bangladesh supports the idea that mortality differentials may be partly attributed to selection for marriage [22].

## Conclusion

Despite modest decreases in adult mortality, the relative survival disadvantage of rural adults persists. A compounding of factors such as rural residence, illiteracy, poor household economic status and women's under-empowerment in household decision making leads to some highly disadvantaged groups in terms of survival. Ameliorating premature adult mortality will require well planned interventions to address factors such as literacy, health service accessibility with special attention to women's needs, socio-cultural changes to improve the status of women and behavioural changes in men.

## Competing interests

The authors declare that they have no competing interests.

## Authors' contributions

MF participated in originating the idea, design of the study, development of the study instrument, overseeing data collection, data analysis, preparing and approval of drafts. YB contributed in designing the study, development of the study instruments, overseeing data collection and reviewing and approving drafts. UH was involved in the design of the study, development of the study instruments, data analysis and reviewing drafts and approval of the final draft. SW participated in originating the idea, designing the study instruments, data analysis and reviewing drafts and approval of the final draft. PB participated in the design of the study, development of the study instrument, data analysis, reviewing drafts and approval of the final draft. All authors read and approved the final manuscript.

## Pre-publication history

The pre-publication history for this paper can be accessed here:


